# Data-driven analysis of neutron diffraction line profiles: application to plastically deformed Ta

**DOI:** 10.1038/s41598-022-08816-7

**Published:** 2022-04-04

**Authors:** Aaron E. Tallman, Reeju Pokharel, Darshan Bamney, Douglas E. Spearot, Bjorn Clausen, Ricardo A. Lebensohn, Donald Brown, Laurent Capolungo

**Affiliations:** 1 Materials Science and Technology Division, Los Alamos National Laboratory, Los Alamos, NM USA; 2 Department of Materials Science and Engineering, University of Florida, Gainesville, FL USA; 3 Department of Mechanical and Aerospace Engineering, University of Florida, Gainesville, FL USA; 4 Theoretical Division, Los Alamos National Laboratory, Los Alamos, NM USA

**Keywords:** Structural materials, Theory and computation, Materials science

## Abstract

Non-destructive evaluation of plastically deformed metals, particularly diffraction line profile analysis (DLPA), is valuable both to estimate dislocation densities and arrangements and to validate microstructure-aware constitutive models. To date, the interpretation of whole line diffraction profiles relies on the use of semi-analytical models such as the extended convolutional multiple whole profile (eCMWP) method. This study introduces and validates two data-driven DLPA models to extract dislocation densities from experimentally gathered whole line diffraction profiles. Using two distinct virtual diffraction models accounting for both strain and instrument induced broadening, a database of virtual diffraction whole line profiles of Ta single crystals is generated using discrete dislocation dynamics. The databases are mined to create Gaussian process regression-based surrogate models, allowing dislocation densities to be extracted from experimental profiles. The method is validated against 11 experimentally gathered whole line diffraction profiles from plastically deformed Ta polycrystals. The newly proposed model predicts dislocation densities consistent with estimates from eCMWP. Advantageously, this data driven LPA model can distinguish broadening originating from the instrument and from the dislocation content even at low dislocation densities. Finally, the data-driven model is used to explore the effect of heterogeneous dislocation densities in microstructures containing grains, which may lead to more accurate data-driven predictions of dislocation density in plastically deformed polycrystals.

The quantification of dislocation content, and of its evolution, during plastic deformation of metals is critical to furthering our understanding of plasticity^1–4^ as well as to the development and validation of microstructure-aware constitutive models^5–7^. Suitably, non-destructive evaluation (NDE) can yield estimates of dislocation densities in bulk^8^. As opposed to microscopy-based methods (e.g., transmission electron microscopy), NDE does not provide a direct observation of defects. Instead NDE measurements are interpreted to identify defect signatures and estimate defect content.

NDE can be performed by either ultrasound spectroscopy^9,10^ or diffraction-based methods^11–13^. With the latter, one utilizes diffraction line profiles or alternatively spatially-resolved lattice strains to estimate dislocation content. The most commonly used method, diffraction line profile analysis (DLPA) has been used to estimate dislocation density in a great number of studies^14–24^. Additionally, much recent work has been done using spatially-resolved lattice strains to characterize microstructure^25–28^. Similarly to the Electron Back Scattering Diffraction (EBSD) method, the crystal orientation and elastic strain fields obtained via High-Energy Diffraction Microscopy (HEDM) (e.g.^25,26^) can be used to estimate the density of geometrically-necessary dislocations (GNDs) density in the medium. Recent techniques use high-resolution EBSD with DLPA models to estimate total dislocation content within a locally resolved volume^27,28^. In all cases, DLPA models analytically relate the shape and breadth of multiple or individual diffraction peaks to the total dislocation density in the bulk^29–34^.

Common to all DLPA methods are several assumptions regarding the geometry of dislocation configurations. Straight, parallel dislocations are assumed by a number of DLPA models ^29,31,35–38^. These idealized networks can differ vastly from complex dislocation structures, as seen in 2-D with transmission electron microscopy^39–41^ or in 3-D with electron tomography^42–44^. Over the past few decades, models accounting for the presence of GNDs and for correlations in the positions of dislocations have been proposed^27,28,35,45,46^. These methods use high-resolution diffraction profiles, and relate the breadth of the peak, asymmetry in the broadening, and the asymptotic description of the tail shape to evaluate dislocation density and many other dislocation arrangement-specific measurements. Modern DLPA methods can further estimate the dislocation content on each slip system. This relies on the use of average contrast factors, which describe the effects of the relative orientation of the dislocation line and of its Burgers vector with respect to the diffraction vector on the magnitude of broadening^47^.

In practice, broadening is also induced by the instrument itself. Thus, using an instrument with high resolution, removing background scattering, and minimizing experimental noise are all critical to the estimation of the defect content^46^. For example, the extended convolutional multiple whole profile (eCMWP) method^11,21^ accommodates instrumental broadening alongside theoretical broadening from defects, to allow dislocation density estimation in the presence of significant instrumental broadening. In the eCMWP approach, an optimization is needed to curve-fit a theoretical profile to the observed profile. This optimization has a sensitivity floor: if strain broadening is small relative to instrument broadening, the optimization cannot be performed. The current work proposes an alternative method to estimate dislocation density from whole diffraction line profiles in the presence of instrumental broadening.

As stated, DLPA provides dislocation content estimates, however quantifying the accuracy of these estimates is non-trivial. Simulations have been proposed as an alternative means of validation for DLPA^28,48^. For example, Balogh et al. simulated single crystal microstructures using discrete dislocation dynamics (DDD)^48^. They employed a simple virtual diffraction approach based on the Stokes-Wilson approximation, calculating broadened peaks directly from strain fields obtained from DDD. They then estimated dislocation density from virtual diffraction profiles with eCMWP to compare with the simulated microstructures. Although this study showed good agreement generally between the dislocation densities extracted from DLPA and generated from dislocation dynamics, when dislocation density varied spatially within a microstructure, significant discrepancies were noted. Nevertheless, both the modest number of cases considered (5) and the approximations made in generating the virtual profiles limited the general applicability of their approach. Since then, several continuum-based methods have been proposed to more accurately generate virtual profiles^49–52^.

Recently, improvements to the speed and accuracy of virtual diffraction algorithms^6,48–53^ and to the efficiency of mesoscale simulations^54^ have enabled the rapid generation of hundreds of diffraction profile-microstructure pairs^50^. For example, Bamney et al.^50^ developed two strain-based virtual diffraction algorithms, one using the Stokes-Wilson approximation^34^, the other using differential strains to include the effects of spatially correlated strains on broadening. In the same work, a data-driven DLPA model, on which the present work is based, was proposed^50^. This data-driven model uses statistical inference to link virtual diffraction profiles to dislocation density, allowing the core DLPA assumptions of idealized microstructures to be relaxed.

The present study builds upon these developments to derive an extended data-driven DLPA model which can be applied to experimentally gathered peaks with an extended sensitivity floor. The proposed approach further provides a quantification of the uncertainty associated with dislocation density estimates. First, synthetic diffraction profile data are generated from simulations of dislocation networks in pure Ta single crystals with DDD by leveraging the two virtual diffraction algorithms proposed in Bamney et al.^50^. Using gaussian process regression (GPR) in an ensemble method, a data-driven DLPA model is built for each diffraction algorithm. The newly proposed methods are employed to estimate dislocation content from experimentally observed neutron time-of-flight (TOF) profiles of Ta. A baseline of predictions is established for the experimental profiles using the eCMWP software^11^. Both of the ensembles provide plausible predictions of dislocation density as a function of applied strain in the presence of experimental noise and instrumental broadening. The method provides uncertainty intervals alongside the estimates. Data-driven estimates are given for experimental profiles with minimal strain broadening for which eCMWP could not give estimates. The extension of the method to include effects of intergranular heterogeneity in plastically deformed polycrystalline Ta is discussed.

## Results

**Figure 1:**
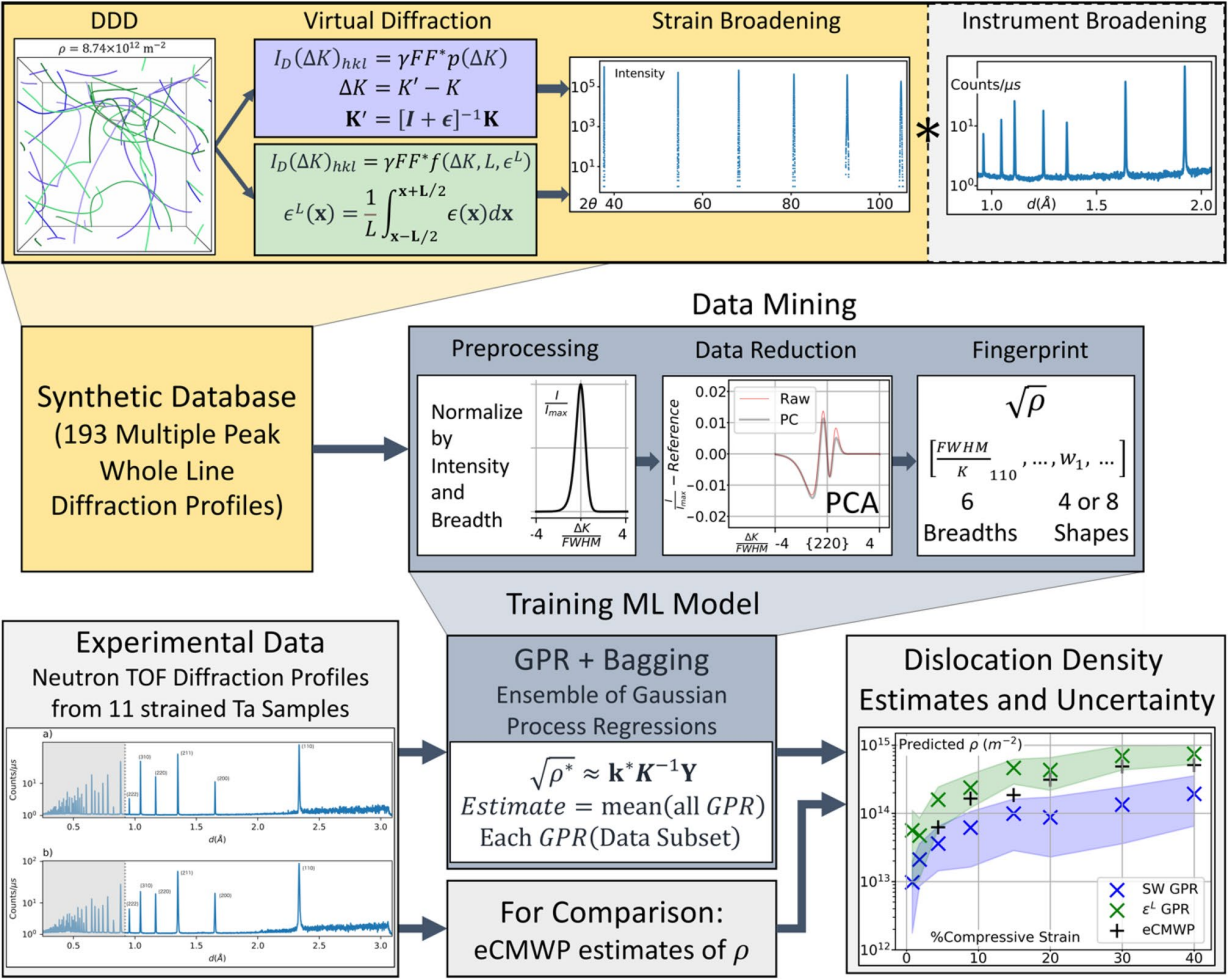
The general outline for the approach, beginning with the synthetic database generation, with discrete dislocation dynamics and virtual diffraction algorithms to calculate strain broadening, combined with instrument broadening obtained from calibration experiments. The data mining procedure is shown, including preprocessing steps, the data reduction using principal component analysis (PCA) and resulting in a fingerprint to be used in a machine learning (ML) model. Lastly, the use of the ML model is shown as a comparison with an existing method (eCMWP) in the prediction of dislocation density from experimental neutron diffraction profiles of deformed Ta samples.

As established in the methodology section, the data-driven estimates of dislocation density originate from a GPR model-ensemble (“Data-Driven Model Form” section), fit to a database of diffraction profiles (Sect. Diffraction profile preprocessing), made by combining synthetically generated strain broadened profiles with experimentally gathered instrumental broadening. The method relies on two main steps: determining a compact description of profile shape, then relating that description to dislocation density. Notably, machine learning techniques (e.g., GPR) are used to produce dislocation density estimates with a data-driven model (“Data-Driven Methods” section), as opposed to an analytical model. The data-driven model is trained on 177 synthetic profiles, incrementally validated against 16 synthetic profiles, and fully validated against 11 experimentally gathered diffraction profiles from plastically deformed Ta samples (gathered as described in “Experiments” section). Two sets of results are obtained, respective to the two algorithms that are used to calculate the strain broadening via virtual diffraction (“Virtual diffraction” section), one without strain correlations (referred to as the Stokes-Wilson-based model) and one with strain correlations (called the ${\epsilon }^{L}$-based model). The entire computational workflow is presented in Fig. 1. Accordingly, the key outcomes of this work are presented in four sections. The synthetic database of microstructures and associated virtual profiles is described in “Database of synthetic profiles” section. The data-driven approximations of the experimental profiles are described statistically in “Approximation of experimental profiles”. The dislocation density predictions of each of the two virtual diffraction algorithms are compared in “Application of the two virtual diffraction algorithms against experimental data” section. The data-driven predictions are compared with the predictions of CMWP in Sect. Application of the two virtual diffraction algorithms against experimental data

### Database of synthetic profiles

**Figure 2:**
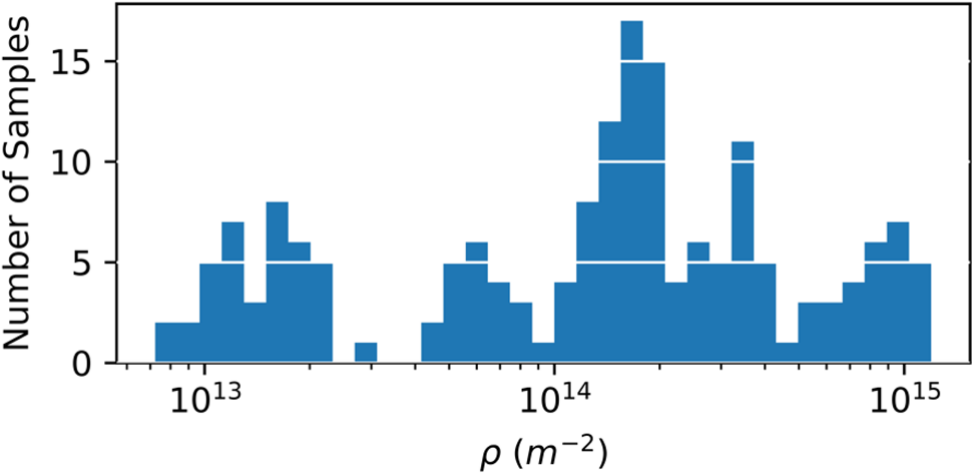
Histogram of dislocation densities in the database of Ta single crystal microstructures generated using DDD.

**Figure 3:**
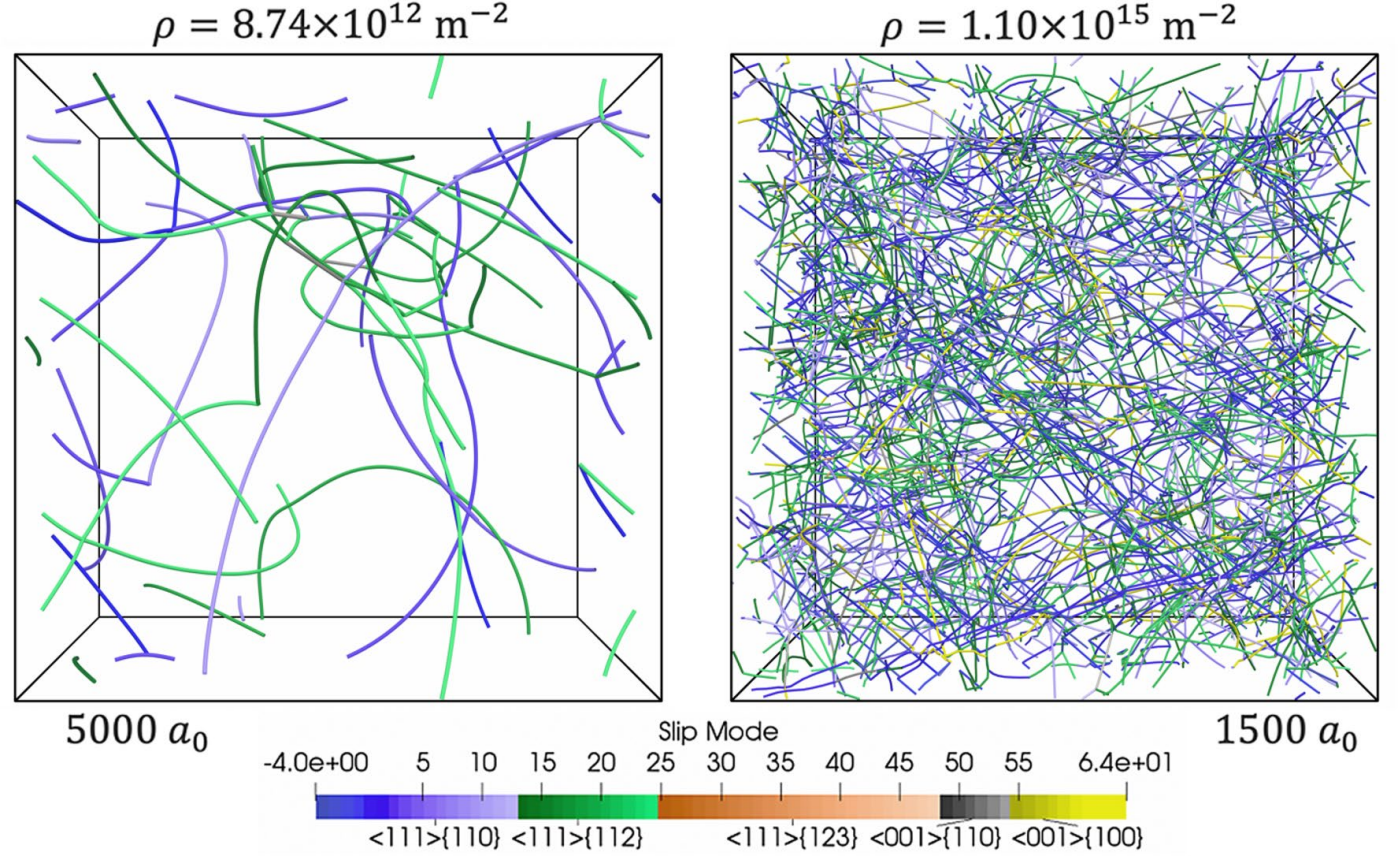
Two simulated microstructures of different dislocation densities, with slip systems labelled by slip mode.

A set of 193 synthetic relaxed microstructures with dislocation densities ranging from $7.23 \times 10^{12}$ to $1.19 \times 10^{15} \;{\text{m}}^{ - 2}$ was generated (see “Data-Driven Methods” section for details of the approach used to generate these profiles). A histogram of the dislocation densities in the dataset is shown in Fig. 2. Two microstructures taken from the database are shown in Fig. 3. The dislocation density $(8.74 \times 10^{12}$ and $1.10 \times 10^{15}$ ${\text{m}}^{ - 2}$) and dimension length ($5000 \cdot a_{0}$ and $1500 \cdot a_{0}$) are provided. Notably, both relaxed dislocation networks shown in Fig. 3 contain dislocations on multiple slip systems, junctions, and curved dislocation lines. The simulated microstructures were not observed to contain dislocation cell structures. A direct comparison between the microstructure generated from DDD and those observed by means of TEM remains challenging. This is due in part to the relaxation of the dislocation structures induced by the sample preparation. In recent work^8^, such dislocation relaxation process was simulated by means of DDD. The study showed that the dislocation content can reduce significantly during preparation of a thin film, and more importantly–that the dislocation lines are very likely to reorient to minimize their image forces. Nonetheless, the dislocation configurations generated via DDD can be compared qualitatively to those documented experimentally–notably to those presented in seminal studies by Spitzig and Mitchell^55,56^. Clearly the synthetic microstructures generated show, as per Fig. 3, that the configurations are characterized by dislocation tangles (see Fig. 3a) of which density increases with overall dislocation content. However, given that cross glide (i.e. cross slip and climb) processes are not activated, the microstructure simulated precludes one from generating cell-like structures with minimum energy configurations^57^. In comparing the dislocation configurations to those reported, in the case of single crystal deformed in the quasi-static regime, it appears that the microstructures generated synthetically mimic those corresponding to stage I and stage II hardening but not of stage III. Thus, the synthetic database is likely to be more representative of microstructures associated with samples deformed up to 20 to 30 percent strain.

Diffraction profiles were calculated from all synthetic microstructures obtained with DDD-FFT, with the intention of capturing experimental diffraction conditions for different configurations. For each dislocation microstructure, the {110}, {200}. {211}, {220} {310}, and {222} peaks were generated numerically using two distinct virtual diffraction algorithms, which include the effect of instrumental broadening. The two algorithms are detailed in “Virtual diffraction” section and are referred to as the S-W-based and $\epsilon^{L}$-based methods. As the first algorithm is computationally much faster than the more accurate second algorithm, an evaluation of predictions using results of both methods are of interest.

**Figure 4:**
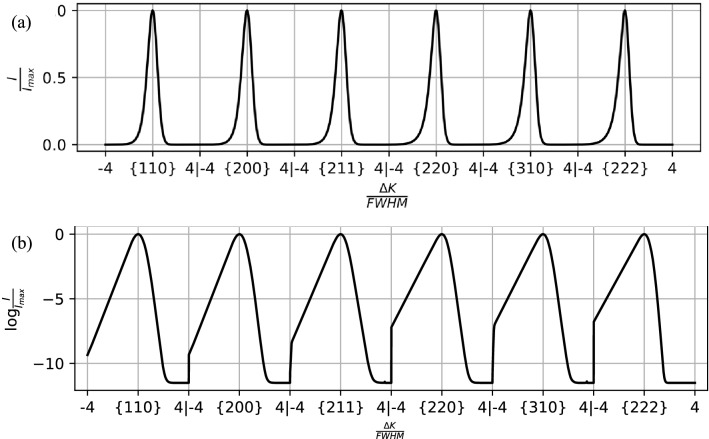
(**a**) An example profile (dislocation density of $8.47 \times 10^{12} {\text{ m}}^{ - 2}$) from the $\epsilon^{L}$-based synthetic database that has been processed to isolate variations in peak shape. (**b**) The profiles shown on a log scale highlight the shape of the tails of the peaks.

The shape variation of each peak is assessed by normalizing the peak intensity *I* by the maximum intensity of the peak $I_{max}$ and plotting these as a function of $\frac{{{\Delta }K}}{FWHM}.$ Here ${\Delta }K$ denotes the magnitude of the difference between the diffraction vector and the ideal diffraction vector as given by Bragg’s condition and FWHM denotes the full width at half maximum. In other words, peaks from the profile were normalized in intensity and peak breadth. Also, the maximum intensity of each peak was shifted to align with ${\Delta }K = 0$. Then, the peaks that were normalized separately were joined into a single processed profile, an example of which is shown in Fig. 4a. The profiles are also shown in Fig. 4b with a log scale to highlight details in shape of the tails of the peaks. A cutoff of four times the $FWHM$ was used to restrict each processed profile to a consistent number of intensity values. Additional processing steps used to remove noise and background from the experimental profiles are detailed in “Diffraction profile preprocessing” section.

### Approximation of experimental profiles

The data-driven compact representation of diffraction profile shape is evaluated in terms of the accuracy of the approximation, which is important to the subsequent predictions. The compact description of the diffraction profile shape (calculated using principal component analysis, PCA) is used to approximate the experimental profiles. The PCA (described in Sect. Diffraction profile preprocessing) is determined using the synthetic profile database (described in Sect. Database of synthetic profiles), and thus may not be as accurate when used to approximate the experiments. The quality of the GPR ensemble predictions (“Data-Driven Model Form” section ) necessarily depend on the extent to which the synthetic profiles resemble the experimental profiles. To compare the datasets, the mean squared error (MSE) was measured for the PCA of profile shape variations across every profile. The statistics describing each dataset are shown in Table 1. The average value and minimum value of MSE across each set of profiles is shown. The training set is the 177 profiles used in the PCA, the synthetic testing set is the 16 synthetic profiles held in reserve, and the experiment set is the 11 profiles obtained from the Ta samples. The training set statistics show the performance of the PCA in an ideal case, establishing a baseline. The synthetic testing set is included to demonstrate the expected increase in error when statistically equivalent profiles outside the training set are considered. The increase in error for the experimental profiles is substantially larger than that for the synthetic testing set, which points to difference between that data and the synthetic data. The difference that these MSE values indicate is quite pronounced and can be addressed further.

**Table 1:** Statistics of the goodness of fit between the PCA representation and the variation of the processed profiles, shown for the two databases. The training set corresponds to the synthetic profiles used to produce the principal components, the synthetic testing set corresponds to synthetic profiles withheld from the PCA, and the experiment set corresponds to the profiles taken from experiments.

Algorithm	Training set of 177 MSE ($\times {10}^{-5}$)		Synthetic testing set of 16 MSE ($\times {10}^{-5}$)		Experiment set of 11 MSE ($\times {10}^{-5}$)	
	$\mathrm{Mean}$	$\mathrm{Max}$	$\mathrm{Mean}$	$\mathrm{Max}$	$\mathrm{Mean}$	$\mathrm{Max}$
S-W	0.0525	0.199	0.0580	0.117	21.8	31.9
$\epsilon^{L}$	0.0617	0.278	0.0683	0.223	17.7	25.6

**Figure 5:**
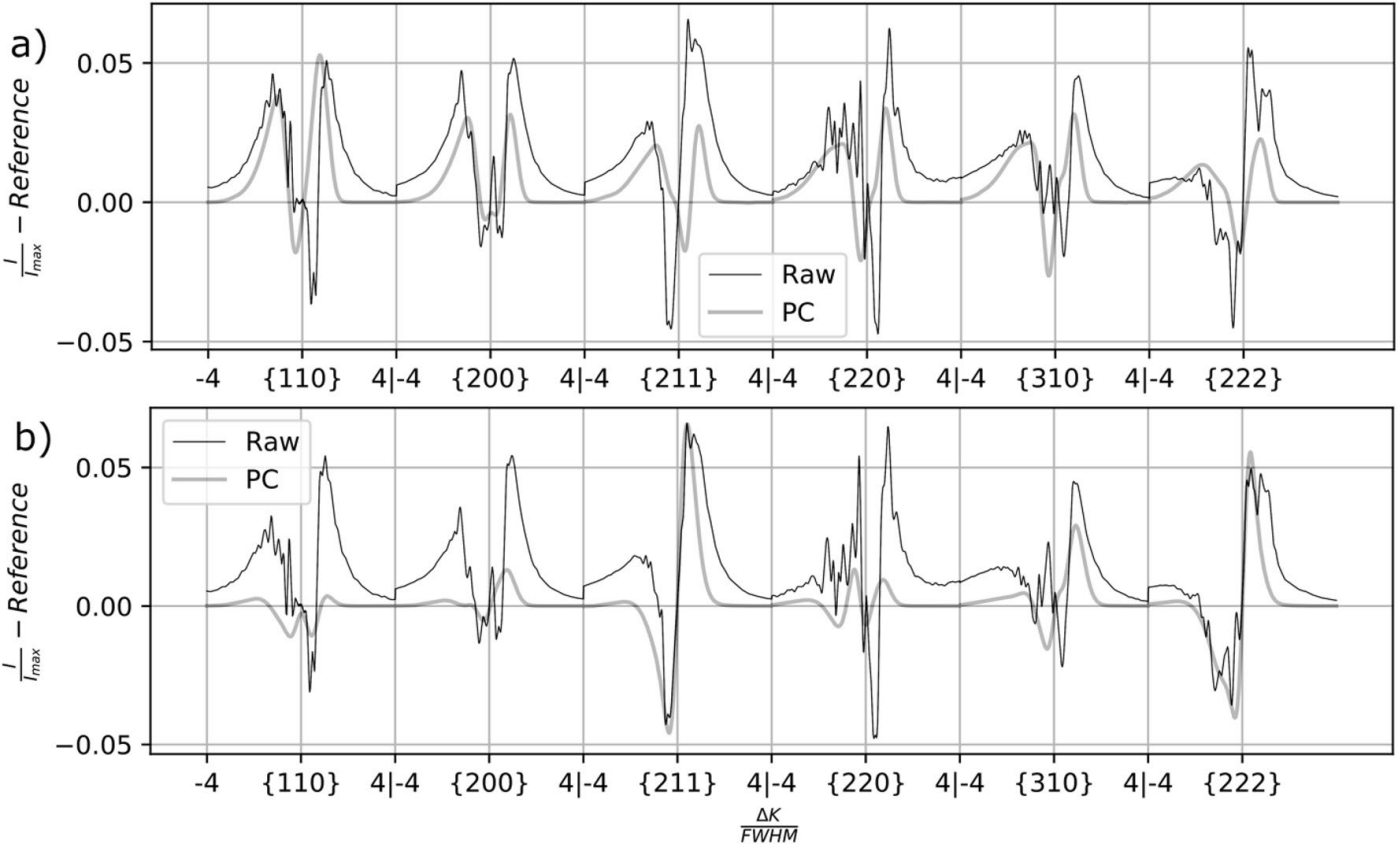
Actual (black) and PC (grey) representation of profile shape variation in the profile from sample IP9.3, based on (**a**) the S-W virtual diffraction algorithm and (**b**) the $\epsilon^{L}$-based virtual diffraction algorithm.

It is not certain whether differences in the experimental and synthetic profiles have bearing on predictions of dislocation density. Differences between the synthetic profiles and the experimental profiles may arise from many sources, such as noise, unremoved background counts, and/or inaccuracy in the single-grain approximation of a polycrystal diffraction profile. These sources may be distinguished by investigating the profiles individually. For example, PCA representations of the experimental profile from one sample are shown in Fig. 5 (sample IP9.3) for both (a) the S-W based dataset and (b) the $\epsilon^{L}$-based dataset. The actual profile shape variation is shown in black, and the PCA representation is shown in grey. The mismatch observed in this case can be summarized as the contributions of noise and of tails which are much broader than those captured by the PCA. While noise is likely unimportant, the discrepancy in the principal component (PC) representation of the tails of the peaks warrants further attention.

### Application of the two virtual diffraction algorithms against experimental data

The data-driven DLPA model was trained with each of the synthetic datasets (S-W and ${\epsilon }^{L}$-based) and used to make predictions of dislocation density from the processed Ta neutron diffraction profiles. Eleven Ta specimens were compressed at 0.001/s strain rate to maximum true strain levels of 0.8, 1.8, 4.5, 9.3, 15, 20, 30 or 40%. Three specimens were loaded with through thickness (TT) and eight with in plane (IP) orientations (“Experiments” section details the experimental method used). The sample information is shown in Table 2 along with the full width at half maximum ($FWHM$) of the $\left\{110\right\}$ peak.

**Table 2:** Names used to refer to the 11 Ta samples are shown alongside the compressive strain at which the respective diffraction profiles were taken. Full width at half maximum measurements are included for the $\left\{110\right\}$ peaks.

Ta sample	IP0.8	IP1.8	IP4.5	IP9.3	IP15	IP20	IP30	IP40	TT20	TT30	TT40
Final % compressive strain	0.8	1.8	4.5	9.3	15	20	30	40	20	30	40
$FWH{M}_{110}\left(d\right)$ $\boldsymbol{\mbox{\AA}} \times 1{0}^{-3}$	4.32	4.13	4.40	5.41	5.32	5.25	5.78	6.64	5.44	5.88	6.20

**Figure 6:**
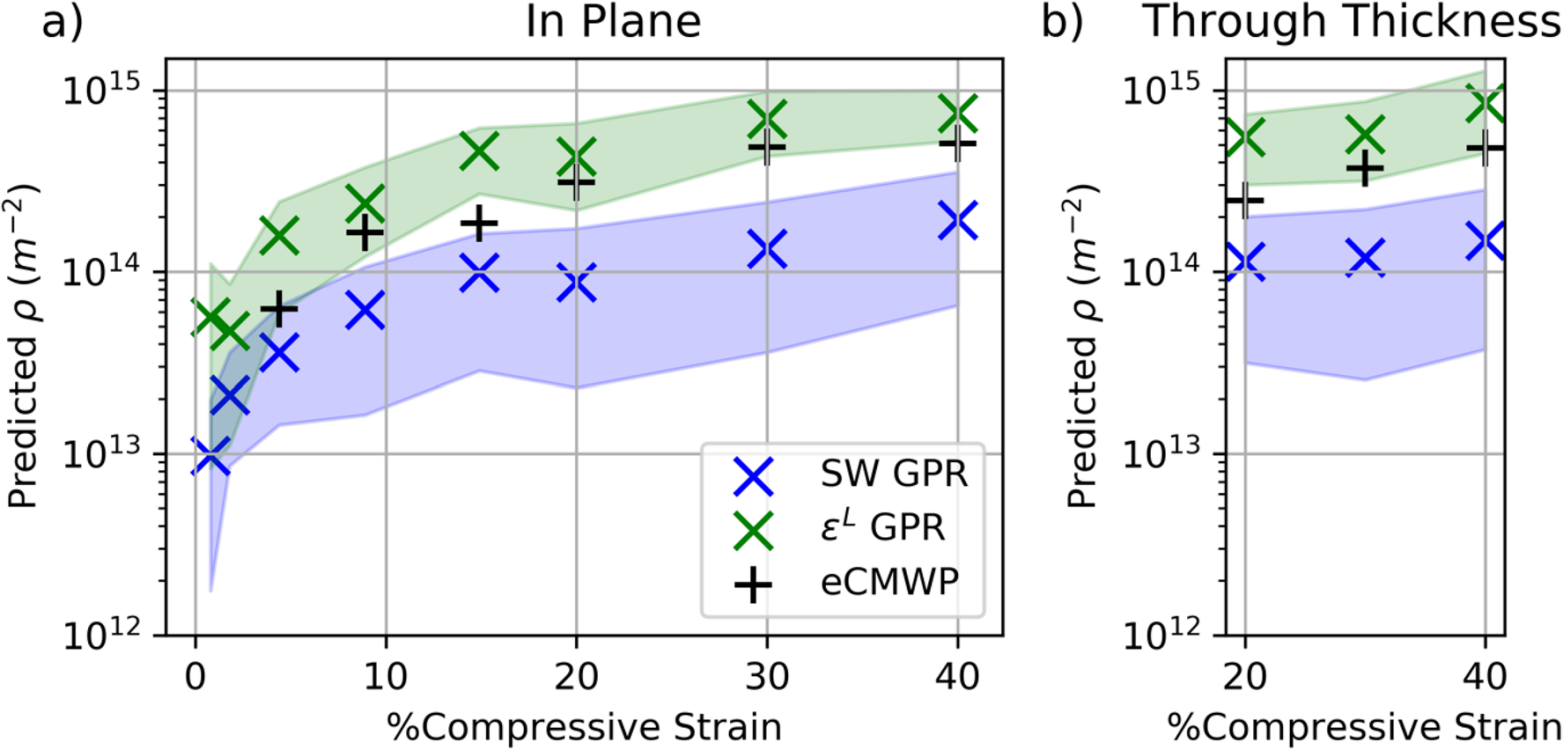
Predictions of dislocation density in the Ta samples from each method, as a function of applied true compressive strain. The shaded regions represent the middle 90% of individual GPR predictions in the ensemble. The eCMWP estimates are shown as a baseline for comparison, where the method was able to produce an estimate. (**a**) Shows the predictions for samples taken in the in-plane direction, and (**b**) shows the samples taken in the through-thickness direction.

Both data-driven DLPA ensemble methods were applied to these 11 experimental profiles. Predictions of the dislocation densities using these two methods along with the baseline made using eCMWP are shown in Fig. 6a, b. The data-driven estimates are accompanied by uncertainty intervals, unlike the estimates from eCMWP (Sect. 3.3). In Fig. 6, the S-W data-driven model predictions are shown in blue, and the ${\epsilon }^{L}$-based model predictions are shown in green. The ensemble approach consists of 80 GPR predictions for each data-driven model (“Data-driven model form” section). In Fig. 6, the ensembles of predictions are simplified to a mean value and the 5th and 95th percentiles of the ensemble predictions. The percentiles are shown as a shaded region around the plotted mean values for each sample. The ensemble variability was very high for each model: $\left(\frac{max}{min}>10\right)$ for the S-W model for every sample and $\left(\frac{max}{min}\sim 5\right)$ for the $\epsilon^{L}$-based model. This suggests the bagging-based estimates (ensemble mean) are more accurate than what could be obtained using all the data to train a single surrogate model, in both cases. Figure 6 shows the dislocation density versus applied strain trends captured by the methods in this study. The trends of dislocation density with strain suggest the new method provides credible estimates of dislocation content.

The baseline prediction of dislocation density made using eCMWP (Sect. 3.3) is also shown in Fig. 6, although for the two samples with the least applied strain, no baseline was available due to the measurement sensitivity limit. In contrast, the data-driven DLPA methods can be used to study dislocation content at the onset of the elasto-plastic transition. The dislocation densities estimated by the S-W surrogate ensemble were lower than the $\epsilon^{L}$-based estimates (with differences of a factor of roughly 2 to 6). This trend is to be expected for a data-driven model trained using data generated using the S-W approximation, which tends to overestimate broadening. Generally, the $\epsilon^{L}$-based ensemble predictions were higher in dislocation density and lower in ensemble variability than the S-W-based ensemble predictions. Notably, in the two cases with least applied strain, the $\epsilon^{L}$-based ensemble variability was substantially higher. This variability indicates a greater degree of uncertainty associated with the estimate, which can explain why the $\epsilon^{L}$-based estimate of specimen IP0.8 (strained to 0.8 percent) could be higher than that of IP1.8 (strained to 1.8 percent).

## Discussion

**Figure 7:**
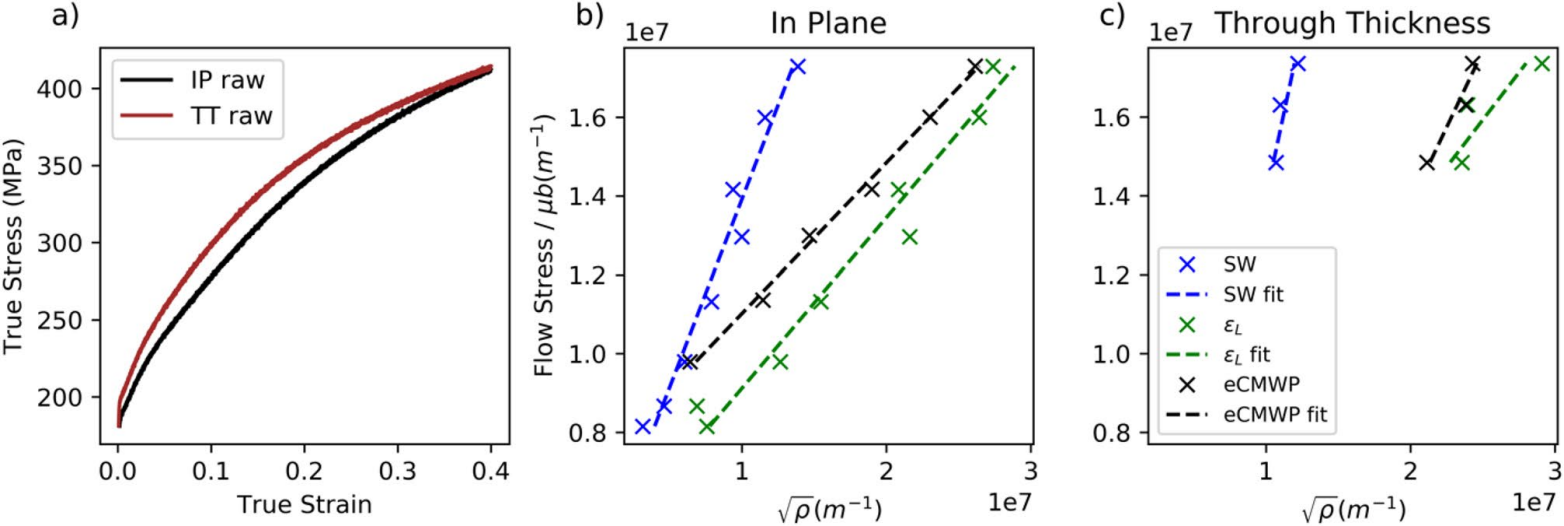
(**a**) Measured mechanical response of Ta during in plane and through thickness loading. Normalized Flow stress vs square root of the dislocation densities for samples loaded in plane (**b**) and through thickness (**c**). The dislocation densities extract by GPR-SW, GPR-$\epsilon_{L}$ and eCMWP are shown in blue, green and black respectively. A linear fit is used to estimate the produce $\alpha M$ in Eq. (1). These fits are shown in dashed lines.

The dislocation densities extracted in “Application of the two virtual diffraction algorithms against experimental data” section can be used to parameterize constitutive models. In its simplest form, the flow stress is related to the dislocation density as per Taylor’s law:

1 $$\sigma_{y} = \alpha M\mu b\sqrt \rho$$

where ${\sigma }_{y}$ denotes the flow stress, $\rho$ refers to the dislocation content while $\mu$ and $b$ refer to the shear modulus and to the magnitude of the Burgers vector. $\alpha$ is typically a fitting constant while *M* is the Taylor factor. The former is representative of the strength of interactions between dislocations while the latter is attributed to texture. Figure 7 shows the measured mechanical response of the system loading along the IP and TT directions as well the relationship between the square root of the dislocation density and the experimentally measured flow stress.

As shown in Fig. 7b, c, irrespectively of the method used to estimate the dislocation density (i.e. SW, or eCMWP), one recovers a linear scaling between the square root of the dislocation content and the normalized flows stress. One further notes that the Taylor expression is expected to break down at large dislocation content, as per studies of Queyreau et al.^58,59^, which suggest that a logarithmic correction should be appended to Taylor’s law at large densities. In the present case though it is found that up to 40% strain leading to estimated dislocation densities greater than 5 × 10^14^ m^−2^ such correction is not necessary. The slopes obtained from a linear fit are for the in-plane loading conditions: GPR-SW: 0.95, GPR-${\epsilon }_{L}$: 0.43, eCMWP: 0.3826. In the case of through thickness loading one obtains: GPR-SW: 1.71, GPR-${\epsilon }_{L}$: 0.47, eCMWP: 0.77. It is thus clear the slopes obtained by the GPR-SW algorithm are far higher than those obtained with GPR-${\epsilon }_{L}$ and eCMWP. Interestingly, it is found that the slopes (i.e. $\alpha M$ in Eq. 1) are found to largely depend on loading direction when using eCMWP. This is not the case when GPR-${\epsilon }_{L}$ is used to extract the dislocation content. Due to the crystal symmetry and materials texture, one does not expect a directional dependence.

From the standpoint of the representativeness of the microstructures used in this study, here single crystal simulations were used to interpret data from polycrystalline samples (“DDD microstructure synthesis” section). In practice though, polycrystalline samples may present variation not captured with the single crystal data. For instance, the single crystal virtual diffraction data could be interpreted as polycrystals of random texture and homogeneous dislocation density between grains, in which grains do not interact. Given that the Ta samples in this study were subjected to plastic deformation and that Ta single crystals are plastically anisotropic, heterogeneity in dislocation density is to be expected between the grains in the Ta samples. In the future, polycrystal DDD simulations may be performed to account for intergranular interactions, and the present analysis may be repeated with the new data.

**Figure 8:**
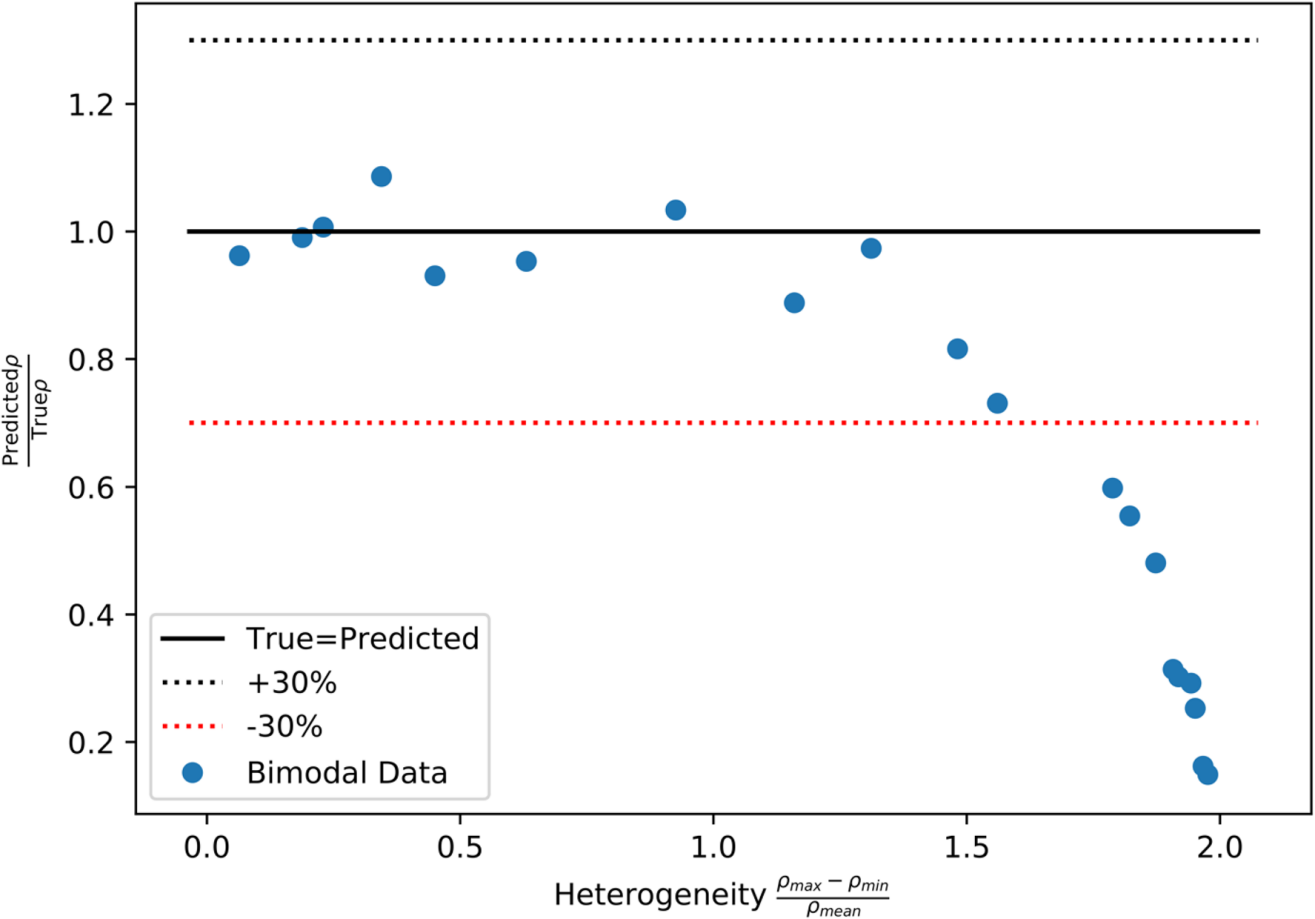
Predictions of the $\epsilon^{L}$-based surrogate model relative to the true values of dislocation density for 20 “bimodal” profiles, as a function of the heterogeneity of the dislocation density between grains.

The current model can estimate error due to the presence of heterogeneity in dislocation density between grains. To demonstrate this, a modification of the synthetic data was performed to develop a new dataset against which the data-driven model could be tested. Two profiles were taken from the synthetic database and combined by adding the intensity as a function of $K$, the diffraction vector length, to each profile. The new profile was used to represent a polycrystal with a bimodal distribution of dislocation densities in different grains. Twenty of these profiles were generated using different pairs of original profiles for each. Grain interactions were still neglected in these new “bimodal” profiles. A parameter *H* was defined to track the heterogeneity in the new profiles as,

2 $$H = \frac{{\rho_{max} - \rho_{min} }}{{\rho_{mean} }}$$

where the values of $\rho_{max}$, $\rho_{min}$, and $\rho_{mean}$ were measured from the two values belonging to the single crystal profiles included in the new profile. The value of $H$ is 0 when the two profiles have the same dislocation density and is 2 when only one profile contains dislocations. A value of $H = 1.64$ reflects an order of magnitude difference between $\rho_{max}$ and $\rho_{min}$. The $\epsilon^{L}$ GPR ensemble was used to predict dislocation density of the “bimodal” profiles, the results of which are shown in Fig. 8. The predicted values divided by true values of dislocation density are shown as a function of $H$. At high levels of heterogeneity, the surrogate model underpredicts dislocation density.

**Figure 9:**
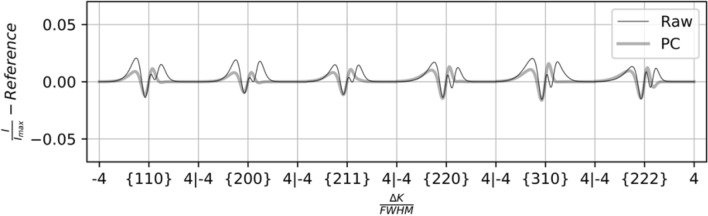
Actual and PC representation of the peak shape variations from a reference peak shape of a bimodal profile with a heterogeneity value of 1.976.

To investigate this further, the PC representation of a bimodal profile is shown in Fig. 8. The bimodal profile has more emphasis in the tails of the peaks, especially for {310}. This can be seen in the third “hump” that is present for all raw peaks, but is not present or is very small for the PC peaks, except for {222}, where it is with opposite sign. The MSE of the PC representation was $2.72 \times 10^{ - 5}$, which falls between the values of the validation data and the experiments. A comparison of the profile shape in Fig. 9 with the profile in Fig. 5 shows that heterogeneity may explain some but not all of the discrepancy between the synthetic database and the experiments.

It is expected that the accuracy of the data-driven model will improve as improvements are made to the database. The number of profiles gathered, the volume of the simulated microstructures, and the types of parameters used in the fingerprint in this work should be improved upon. A study of database size effects on predictions could help to establish database size requirement guidelines. Additional parameters could be used to capture the peak shifts omitted in the current model. Critically, any improvements to the database provide an avenue for improvements to the predictions of the data-driven model. In future work, the method could be extended to predict dislocation content in polycrystals with high contrast in dislocation content between grains.

## Methods

### Experiments

**Figure 10:**
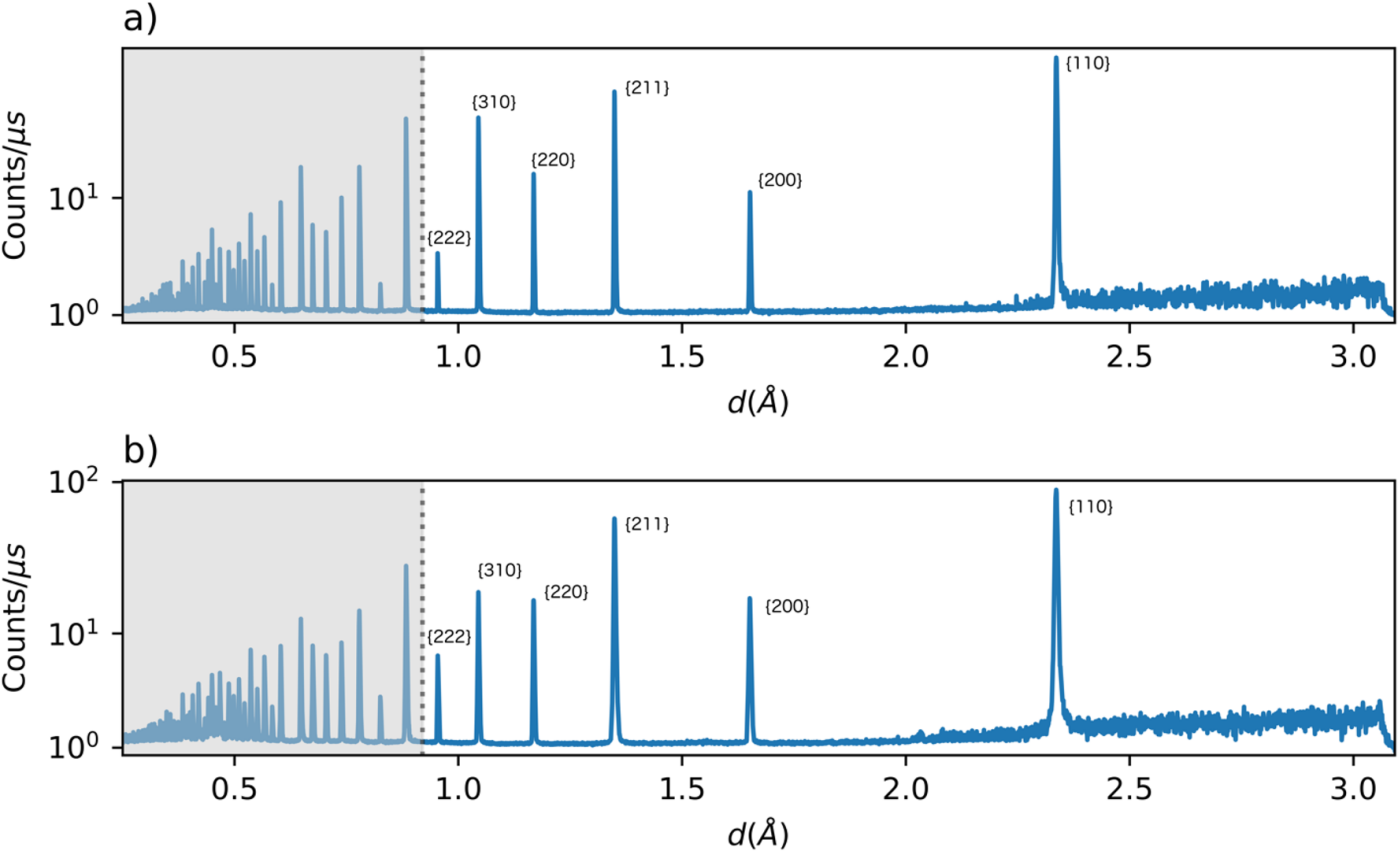
Neutron diffraction profiles measured for IP1.8 (**a**) and IP30 (**b**) are shown. The first six peaks are labelled with the hkl of the diffraction plane. The portion of the profile not used in the analysis is greyed out.

Multiple Ta specimens of 4.2 mm diameter and 8.4 mm height with their axes parallel to the through thickness (TT) and in-plane (IP) directions were electro-discharge machined from a wrought plate. The wrought plate material had an average grain diameter of 30 µm, with nearly equiaxed grains^60^. Eleven Ta specimens (three with TT and eight with IP orientations) were compressed at 0.001/s strain rate to maximum true strain levels of 0.8, 1.8, 4.5, 9.3, 15, 20, 30 or 40%. The diffraction line profile data were recorded on a high-resolution ($FWHM$ ∼ 0.1%) backscattering (153 deg) detector bank on the spectrometer for material research at temperature and stress (SMARTS)^61^. The beam width was 10 mm and the beam height was 5 mm, illuminating nearly all of the sample volume, which was about 100 mm^3^. The diffracted signal was counted for six hours, to ensure high signal to noise ratio. Line profile data were collected for all deformed samples compressed to various levels of final strains. The six peaks with largest interplanar spacing were selected for analysis: {110}, {200}. {211}, {220} {310}, and {222}. Two profiles are shown for reference in Fig. 10. In Fig. 10, the relative intensity of the peaks to the background and noise is shown to be high, and the breadth of peaks was variable between samples. The increase in peak breadth was apparent with increasing macroscopic strain. The peak broadening was asymmetrical for many of the peaks analyzed.

### Data-driven methods

#### DDD microstructure synthesis

Discrete dislocation dynamics (DDD) simulations represent a dislocated lattice as discrete dislocation lines resolved within a continuous elastic medium. The costliest computations of DDD are the solution of dislocation segment velocities and the solution of the lattice stress and strain within the simulated volume. The second part can be accelerated by using a fast Fourier transform (FFT) and periodic boundary conditions (instead of a finite element solver) to solve for the stress field^54,62^. These algorithmic advances increase the effective time-scale and length-scale of DDD simulations. Here, DDD simulations in the FFT-based framework (DDD-FFT)^54,62^ were used to generate microstructures and elastic strain fields associated with relaxed dislocation networks in Ta single crystals. Elastic properties of Ta were taken from experiments reported in^63^. Dislocation motion was assumed to be isotropic and planar. Similar to BCC Fe^64^, slip modes {110}, {112}, and {123} were considered. Further junctions on {100} and {110} were also considered. The parameters used are shown in Table 3. Note that the choice of drag coefficients in Table 3 has little to no influence on the low energy configuration resulting from relaxation. Further, choice was made to set a similar friction stress for all slip mode. This assumption is consistent with recent crystal plasticity based constitutive modeling assumptions in^65^. Note that the values of friction stress utilized in the present study are in the same order of magnitude as those used in^66^ where the authors used values ranging from 27 to 37 MPa. The values of the friction stress for junctions was arbitrarily chosen. This choice is known to impact the length of junctions. The impact of this choice on the overall diffraction signature is not expected to be significant but has not been quantified rigorously.

**Table 3:** The material parameters used in the DDD simulations of Ta.

Slip mode	Peierls Stress (MPa)	Drag (${10}^{-6}$ Pa · s)	Elasticity coefficients	
<111> {110}	10	$80$	${C}_{11}$(GPa)	260.91
<111> {112}	10	$80$	${C}_{12}$	157.43
<111> {123}	10	$80$	${C}_{44}$	81.82
<001> {110}	300	$800$	${a}_{0}$(Å)	3.37
<001> {100}	300	$9000$		

A 64 × 64 × 64 grid of regularly-spaced points where the stress field is computed by means of the FFT-based algorithm was used for all simulations. The size of the simulated volume was adjusted from $1500a_{0}$ to $5000a_{0}$ per side to gather a wide range of dislocation densities while providing at least a minimum number of segments. A minimum of 4000 segments in each microstructure was ensured to reduce the effect of the small sample size. Every microstructure generated in this work contained junctions and dislocations on multiple slip systems.

Dislocations were initialized by distributing randomly positioned elliptical shear dislocation loops on each {110} type slip system. The number of dislocations per slip system on average varied per simulation between 0.8 and 4, with random draws being used to resolve fractions into whole number outcomes. For example, an average value of 1.2 was instantiated with a random selection for each slip system, with one dislocation on a slip system at an 80% chance and two dislocations for the remaining 20%. Dislocations were allowed to reach {112} and {123} type slip systems via cross-slip. A two-step process was used to generate equilibrated dislocation microstructures. First, a high stress was applied to induce loop growth for approximately $1 \cdot 10^{ - 10} \;{\text{s}}$, with the following imposed stress tensor,

3 $${\varvec{\sigma}} = \left[ {\begin{array}{*{20}c} 2 & 1 & { - 1} \\ 1 & { - 3} & { - 2} \\ { - 1} & { - 2} & 4 \\ \end{array} } \right]\user2{ }{\text{GPa}}\user2{ }$$

The shock-level loading led to junction formation as the loops grew and intersected. Second, the system was allowed to relax under internal stresses for $7 \cdot 10^{ - 10} \;{\text{s}}$. This allowed the strain energy of the system to reduce and dislocations to adopt a stable configuration. Notably, the simulated loading has no similarity to the load applied to the experimental samples. The only importance of this stress is that it activates slip on all slip systems. Neither the loading nor the crystal orientation is randomized. Rather, the resulting relaxed dislocation networks have randomness from their initial dislocation loop locations and not from their loading. No attempt was made to generate networks associated with any particular loading history.

#### Virtual diffraction

In the generation of synthetic profiles, broadening contributions from the instrument and from dislocations were included. With this, the diffraction intensity $I\left( {{\Delta }K} \right)_{hkl}$ is related to ${\Delta }K$ as follows:

4 $$I\left( {{\Delta }K} \right)_{hkl} = I_{instr} \left( {{\Delta }K} \right)_{hkl} *I_{D} \left( {{\Delta }K} \right)_{hkl}$$

for each $hkl$ in the six considered peaks (i.e., {110}, {200}. {211}, {220} {310}, and {222}). Size broadening was assumed to be negligible for the single crystal Ta samples in this work.

The virtual diffraction algorithms used here are taken from the work of Bamney et al.^50^. A detailed description of both algorithms is contained in the original work. Here, a brief summary is provided. Both algorithms determine diffracted intensity as a function of diffraction angle using the elastic strain fields calculated using DDD-FFT.

The first algorithm employs the Stokes-Wilson^34^ approximation and determines the broadening of a diffraction peak using a probability distribution of apparent strain, i.e., the elastic strain tensor projected along the direction of the diffraction vector. For a hkl-specific diffraction vector ${\mathbf{K}}$, the intensity of the diffracted radiation can be defined as

5 $$I_{D} \left( {{\Delta }K} \right)_{hkl} = \gamma FF^{*} p\left( {{\Delta }K = K^{\prime} - K} \right),$$

where $I_{D}$ is the intensity of the diffracted beam, $\gamma$ is the Lorentz-polarization factor, $F$ is the structure factor of the unit cell with complex conjugate $F^{*}$, and $p$ is the probability that the strained lattice will produce a given diffraction vector ${\mathbf{K^{\prime}}}$. The diffraction vector for the strained lattice is calculated as

6 $${\mathbf{K^{\prime}}} = \left[ {{\varvec{I}} + \epsilon } \right]^{ - 1} {\mathbf{K}},$$

with second rank identity tensor ${\varvec{I}}$, elastic strain tensor $\epsilon$, and diffraction vector associated with the unstrained lattice, ${\mathbf{K}}$. The values of $\gamma$ and $F$ are calculated from the structure of the Ta unit cell and the value of ${\mathbf{K}}$. The algorithm approximates $p$ using the value of elastic strain at the $N_{x}^{quad} \times N_{y}^{quad} \times N_{z}^{quad}$ quadrature points contained in each DDD simulation. Calculations are performed for every plane or $hkl$ selected for diffraction. In this work, all planes in a family are assumed to contribute in equal weight to the 1-D profile.

The second virtual diffraction algorithm includes correlations in strain. When lattice strains are correlated across different distances, i.e., at different values of correlation length $L$, the effect of those strains on diffraction peak broadening are diminished. In the previous algorithm these correlations are neglected, which can lead to overestimates of strain broadening. In this method, the diffracted intensity is calculated as,

7 $$I_{D} \left( {{\Delta }K} \right)_{hkl} = \gamma FF^{*} \mathop \sum \limits_{L = - \infty }^{ + \infty } \left[ {A^{D} \left( {L,\epsilon^{L} } \right) + iB^{D} \left( {L,\epsilon^{L} } \right)} \right]{\text{exp}}\left[ {2\pi iL\Delta K} \right],$$

where $\epsilon^{L}$ is the differential elastic strain resolved along the diffraction vector, and the terms $A^{D}$ and $B^{D}$ are the symmetric and asymmetric components of strain broadening, and are defined as,

8 $$A^{D} \left( {L,\epsilon^{L} } \right) = \mathop \smallint \limits_{ - \infty }^{ + \infty } p\left( {\epsilon^{L} } \right)\cos \left( {2\pi LK\epsilon^{L} } \right)d\epsilon^{L}$$

9 $$B^{D} \left( {L,\epsilon^{L} } \right) = \mathop \smallint \limits_{ - \infty }^{ + \infty } p\left( {\epsilon^{L} } \right)\sin \left( {2\pi LK\epsilon^{L} } \right)d\epsilon^{L} .$$

The value of $p\left( {\epsilon^{L} } \right)$, the distribution of differential strain for a specific correlation length $L$, as projected onto ${\mathbf{K}}$, was calculated for each correlation length $L$, which can be gathered from the simulated microstructure ($L$ is bounded by the separation between quadrature points and the distance between the periodic boundaries). The distribution $p\left( {\epsilon^{L} } \right)$ was determined using measurements of differential strain at every quadrature point, ${\mathbf{x}}$ as,

10 $$\epsilon^{L} \left( {\mathbf{x}} \right) = \frac{1}{L}\mathop \smallint \limits_{{{\mathbf{x}} - {\mathbf{L}}/2}}^{{{\mathbf{x}} + {\mathbf{L}}/2}} \epsilon \left( {\mathbf{x}} \right)d{\mathbf{x}},$$

where the integration was performed using a trapezoid rule. Given that the line of ${\mathbf{L}}$ typically does not coincide with the array of quadrature points, a linear interpolation was used to determine the projected strain values along ${\mathbf{L}}$ used in the integration scheme. These calculations were carried out for each $hkl$ selected for measurement.

The strain broadened synthetic profiles were convoluted with a theoretical instrumental broadening function, $H\left( {{\Delta }t} \right)$. The Cu foil profile was used to calibrate the first theoretical instrumental broadening function from the crystallographic software package General Structure and Analysis System (GSAS) for neutron TOF measurements^67^, i.e.,

11 $$H\left( {{\Delta }t} \right) = \smallint G\left( {{\Delta }t - \tau } \right)E\left( \tau \right)d\tau$$

12 $$E\left( \tau \right) = \left\{ {\begin{array}{*{20}c} {2Ne^{\alpha \tau } , \tau < 0} \\ {2Ne^{ - \beta \tau } , \tau > 0} \\ \end{array} } \right.$$

13 $$G\left( {{\Delta }t} \right) = \frac{1}{{\sqrt {2\pi \sigma^{2} } \exp \left[ { - \frac{{\left( {{\Delta }t - \tau } \right)}}{{2\sigma^{2} }}} \right]}}$$

where $N$ is a normalization constant. $N$ is defined,

14 $$N = \frac{\alpha \beta }{{2\left( {\alpha + \beta } \right)}}$$

and where $\alpha , \beta , \mathrm{and} \sigma$ are functions of interplanar spacing, $d$. These functions are determined as interpolations from the values obtained from the calibration profile,

15 $$\alpha = f_{\alpha } \left( d \right),\;\beta = f_{\beta } \left( d \right),\;\sigma = f_{\sigma } \left( d \right)$$

and the observed values used to define these interpolations are shown in Table 4. The values of $\alpha , \beta , \;{\text{and}}\;\sigma$ and the corresponding values of $d$ were obtained using a numerical fitting to the calibration Cu profile.

**Table 4:** Instrument broadening parameters as evaluated with a Cu calibration profile.

hkl	$d$(Å)	$\alpha$	$\beta$	${\sigma }^{2}$
111	2.08708	0.20804	0.045725	328.93
200	1.80743	0.26294	0.050833	254.44
220	1.27831	0.17400	0.054888	113.39
311	1.09013	0.23225	0.056578	76.323
222	1.04373	0.27436	0.061723	72.076
400	0.904023	0.14311	0.051810	18.493
331	0.829475	0.32658	0.058277	36.100
420	0.808477	0.33464	0.059525	36.752
422	0.738017	0.43557	0.060113	33.542
511, 333	0.695886	0.28550	0.063623	18.795

To convert $H\left( {{\Delta }t} \right)$ from TOF microseconds to small difference vector ${\Delta }K$, the following relation was used,

16 $$T_{ph} = DIFC d + DIFA d^{2} + ZERO,$$

where $DIFA$, $DIFC$ and $ZERO$ are known calibration quantities for the instrument. Using this, $H$ can be expressed in terms of constants and ${\Delta }K,$

17 $$I_{instr} \left( {{\Delta }K} \right)_{hkl} = H\left( {\frac{DIFC}{{K_{hkl} + {\Delta }K}} + \frac{DIFA}{{\left( {K_{hkl} + {\Delta K}} \right)^{2} }} + ZERO - T_{ph} \left( {d_{hkl} } \right)} \right)$$

#### Diffraction profile preprocessing

**Figure 11:**
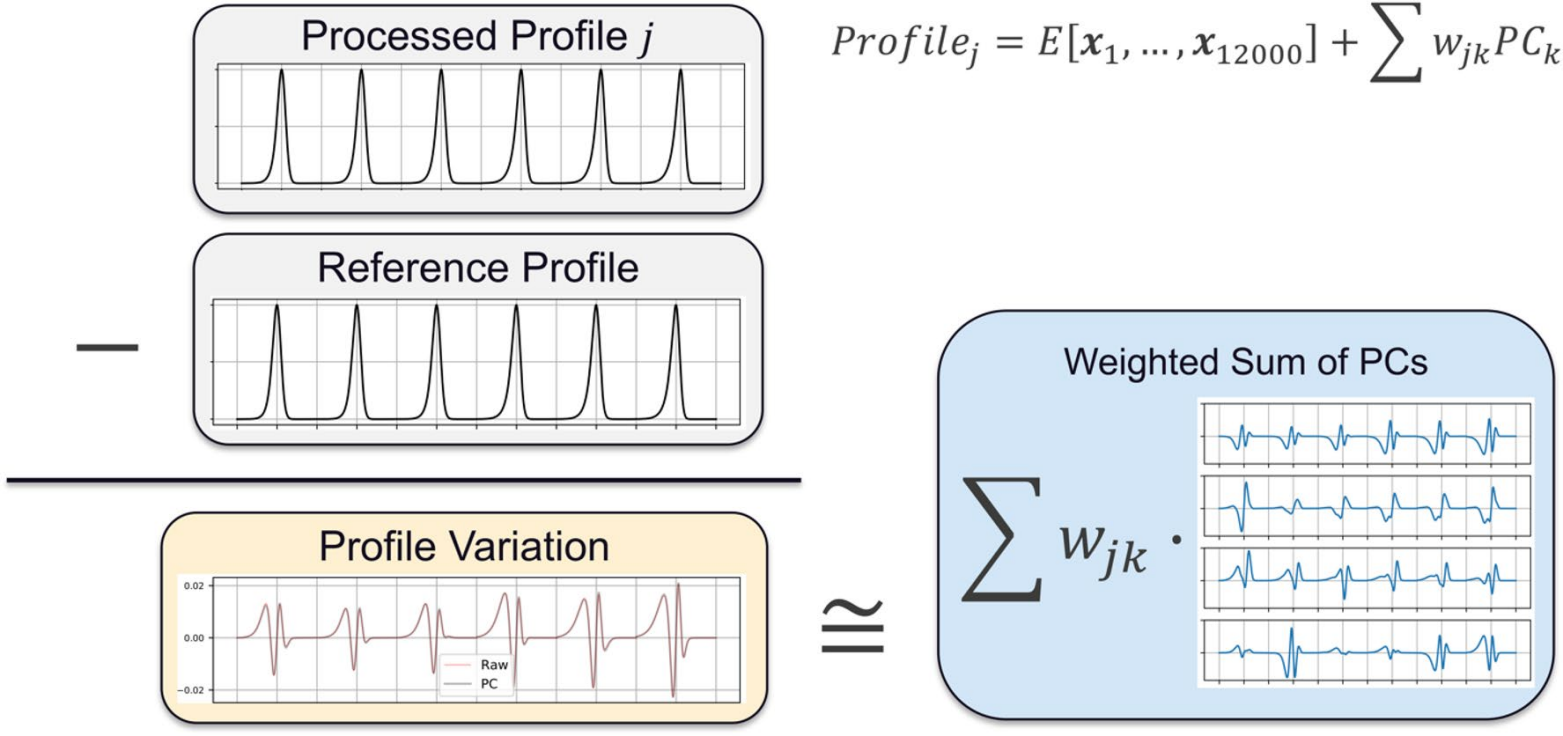
Schematic of how the principal component analysis reproduces a processed profile from principal components.

PCA was used to obtain a quantitative description of peak shape variations with a minimal set of values. This procedure is given as a schematic in Fig. 11. The PCA was applied to the database of processed synthetic profiles. At each of the 12,000 values of ${\Delta }K/FWHM$, the $\frac{I}{{I_{{max{ }}} }}$ values of each of the set of profiles is expressed as a vector,

18 $${\varvec{x}}_{i} = \left[ {\frac{I}{{I_{{max{ }}} }}\left( {\frac{{{\Delta }K}}{{FWHM_{i} }}} \right){\text{for}}\;{\text{all}}\;{\text{profiles}}\;{\text{in}}\;{\text{set}}} \right],\;\;{\text{for }}i \in \left[ {1,\;12,000} \right]$$

**Figure 12:**
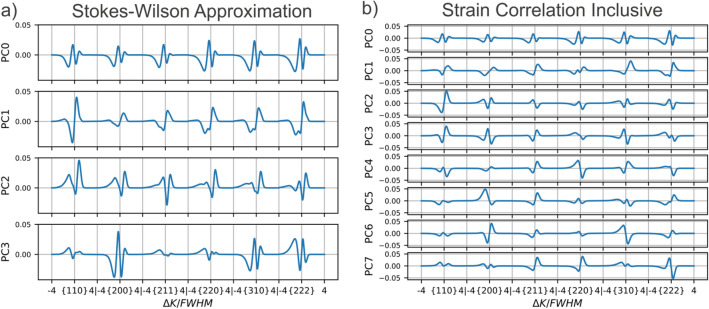
The principal components (PCs) which were used to approximate profile shape for the two different profile databases. The components are given in order of decreasing relative contribution to the total variance of the dataset.

Eigenvectors are obtained for the covariance matrix of ${\varvec{x}}$,

19 $$cov\left[ {{\varvec{x}}_{i} ,{\varvec{x}}_{j} } \right] = E\left[ {\left( {{\varvec{x}}_{i} - E\left[ {{\varvec{x}}_{i} } \right]} \right)\left( {{\varvec{x}}_{j} - E\left[ {{\varvec{x}}_{j} } \right]} \right)} \right],$$

where $E\left[{{\varvec{x}}}_{i}\right]$ is the expected value (i.e., mean) of ${{\varvec{x}}}_{i}$. The eigenvectors with the largest corresponding eigenvalues become the PCs. Once obtained, the PCs can approximate any profile as a weighted sum, i.e.,

20 $$Profile_{j} = E\left[ {{\varvec{x}}_{1} ,{ } \ldots ,{\varvec{x}}_{12000} } \right] + \sum w_{jk} PC_{k} ,$$

where $E\left[ {{\varvec{x}}_{1} ,{ } \ldots ,{\varvec{x}}_{12000} } \right]$ is the mean of the set of profiles (i.e. a reference profile). Using the same set of PCs, unique weight vectors, ${\varvec{w}}_{j}$, were found for all the profiles in the available set of 177 (16 of the original 193 profiles were reserved for validation). The weight vector, ${\varvec{w}}_{j} = \left[ {w_{0} , \ldots ,w_{k} } \right]_{j}$, associated with each profile is a compressed, approximate, and quantitative signature of the peak shapes. The weights therefore contain the measurable effects of the variation in dislocation density and arrangement present in the database. More than 97.5% of the variation in the $\epsilon^{L}$-based dataset was captured using 8 PCs, and over 99% of the variation in the S-W dataset was captured using 4 PCs. The PCs are shown in Fig. 12. The PC weights used to approximate each profile were used as the description of peak shape given to the surrogate model.

The compressed data was organized in a “fingerprint” for each profile. The fingerprint contains the $\frac{FWHM}{K}$ of each peak. The series of weights which can reproduce the shape of the profile from the PCs were also included in the fingerprint. The square root of the dislocation density was selected as the dependent variable of the surrogate model. Values of $FWHM$ are expected to vary with $\sqrt \rho$^31^. The data used by the surrogate model amounts to a compact vector of quantities,

21 $$\begin{aligned} & Output: \sqrt {\rho_{i} } \\ & Input: \left[ {\frac{FWHM}{K}_{110} ,\frac{FWHM}{K}_{200} ,\frac{FWHM}{K}_{211} ,\frac{FWHM}{K}_{220} ,\frac{FWHM}{K}_{310} ,\frac{FWHM}{K}_{222} ,w_{0} ,w_{1} ,w_{2} ,w_{3} \left( {, \ldots } \right)} \right]_{i} \\ \end{aligned}$$

where the subscript $i$ indicates the $i$-th profile in the dataset. The PCA weights $w_{0}$, etc., continue to $w_{7}$ for the fingerprints associated with the $\epsilon^{L}$-based dataset.

#### Data-driven model form

The data-driven model uses Gaussian process regression (GPR) surrogate models in an ensemble. A GPR uses the covariance between fingerprints, ${\mathbf{x}}_{i}$, in the database, ${\varvec{X}}$, to make predictions for new cases based on the observed dislocation densities and covariance matrix ${\varvec{K}}$. These predictions can be made after the GPR has been trained, i.e., numerically fitted to a portion of data designated for training the model. The quantity of interest of a new case, $y^{*} = \sqrt {\rho^{*} }$, is then predicted using the mean value prediction $\hat{y}^{*}$ (where $y^{*}$ may be drawn from a Gaussian distribution with mean = $\hat{y}^{*}$), i.e.,

22 $$\hat{y}^{*} = {\mathbf{k}}^{*} \left( {{\varvec{K}} + \alpha {\varvec{I}} } \right)^{ - 1} {\mathbf{y}},$$

where ${\mathbf{y}}$ is the vector of observed values of $y$ in the training data, and ${\mathbf{k}}^{*}$ is the vector of covariances between the new fingerprint ${\mathbf{x}}^{*}$, and the fingerprint matrix ${\varvec{X}}$. ${\varvec{I}}$ is the identity matrix and $\alpha$ is a tunable non-negative quantity which accommodates noise in ${\mathbf{y}}$. The covariance between fingerprints is defined using a kernel function. This function provides a means of tuning the covariance between two fingerprints, ${\mathbf{x}}_{i}$ and ${\mathbf{x}}_{j}$, as a function of the distance $d$ (Euclidean or otherwise) between them. The kernel functions used in this work are given as,

23 $$k\left( {d\left( {{\mathbf{x}}_{i} ,{\mathbf{x}}_{j} } \right), {{\varvec{\uptheta}}}} \right) \in \left\{ {k_{Matern} ,k_{RBF} ,k_{RQ} } \right\}$$

where ${{\varvec{\uptheta}}}$ is a vector of tunable values called hyperparameters and the various kernel functions are defined in Table 5. The chosen kernel function form was the best-fitting to the data, or specifically the highest in terms likelihood of the data, given the optimized hyperparameters and kernel function. The algorithm of the training of GPR models can be found in^68,69^.

**Table 5:** Kernel function forms which are tested to determine the best performing alternative.

Kernel Function Forms
$$k_{RBF} = \exp \left( { - \frac{1}{2}\left( \frac{d}{l} \right)^{2} } \right), {{\varvec{\uptheta}}} = \left[ l \right]$$
$$k_{Matern} \left( {\nu = 1.5} \right) = \left( {1 + \frac{\sqrt 3 d}{l}} \right)\exp \left( { - \frac{\sqrt 3 d}{l}} \right),{{\varvec{\uptheta}}} = \left[ {l,\nu } \right]$$
$$k_{Matern} \left( {\nu = 2.5} \right) = \left( {1 + \frac{\sqrt 5 d}{l} + \frac{{5d^{2} }}{{3l^{2} }}} \right)\exp \left( { - \frac{\sqrt 5 d}{l}} \right)$$
$$k_{RQ} = \left( {1 + \frac{{d^{2} }}{{2\phi l^{2} }}} \right)^{ - \phi } , {{\varvec{\uptheta}}} = \left[ {l,\phi } \right]$$

The GPR was applied in an ensemble approach that uses many random subsamples of 142 of the 177 training profiles to develop 80 GPR models from which an average is taken. The method is known as bagging (from bootstrap-aggregating)^70,71^. This method is recommended in cases where the predictions of a statistical model (here GPR) are subject to large variations when different training data are used. To ascertain if this was the case here, the variation in the bootstrapped GPR predictions was inspected. Where this variation is large, the use of bagging is known to increase the accuracy of the overall predictions. The variation is presented alongside the ensemble predictions in “Application of the two virtual diffraction algorithms against experimental data” section. The variability of the ensemble predictions is also used as an indicator of the uncertainty in the estimates. It is noted that each GPR also produces an estimate of variance with each prediction, however these were not used in this analysis.

### LPA comparison methods

A baseline set of dislocation density predictions were obtained using existing LPA methods. Semi-quantitative analysis of the microstructural features responsible for the increase in peak breadth was performed using a whole diffraction pattern modeling method implemented in the extended CMWP (eCMWP) software^11^. The formulation of strain broadening used in eCMWP is outlined in “Virtual diffraction” section.

Instrumental broadening was taken from the diffraction of a strain and dislocation free NIST standard Si powder sample. Typically, TOF neutron diffraction data show sharp leading and broad trailing tail-shapes; therefore, a Pearson VII function was used to fit the two sides of the profile to obtain separate breadth and shape parameters for each side of each peak. Linear fits for both shape and breadth versus $K$ were used to calculate the instrument profile at an arbitrary $K$ position. Blanket background subtractions were performed on all diffraction profiles as a pre-processing step. Finally, the calculated whole diffraction pattern was fitted to the measured pattern to refine the microstructural parameters.

A Levenberg–Marquardt algorithm is used to estimate fitting parameters that are used to calculate the arrangement and density of dislocations. Table 6 shows the material parameters used for Ta to determine initial guesses for values needed prior to optimization, specifically the dislocation character parameter ($q$) and average contrast factor from Ungar et al.^72^. These initial guesses were $q_{av} = 0.5$, assuming 100% edge type dislocations, and $C_{h00 - av} = 0.2$^11^. Estimated values were obtained for area weighted coherently diffracting domain size, dislocation density, dislocation arrangement, and dislocation character. The effects of texture were not included in this analysis. Note, the experimental dislocation densities in as-received Ta and deformation < 5% strain were below the measurement sensitivity and are not reported here.

**Table 6:** Parameters used in the calculation of the contrast factor and dislocation character as required in eCMWP.

Slip Modes	${C}_{11}(\mathrm{GPa})$	${C}_{12}$	${C}_{44}$	${a}_{0}$ (nm)	$b$ (nm)	Zener const. (${\mathrm{A}}_{\mathrm{z}}$)	$\frac{{C}_{12}}{{C}_{44}}$
<111> {110} <111> {112}	262.77	160.88	81.44	0.33026	0.28601	1.5986	1.9754

### Experiment data preprocessing

To accommodate the data-driven method, background counts and noise were removed from the experimental profiles. Rietveld refinement is commonly used, however the assumption of symmetry in strain broadening is problematic for the whole profile data-driven analysis (that makes no such assumption). A heuristic-based method was developed to avoid biasing the data. The background was subtracted by fitting a Gaussian process to data points manually selected from the background.

**Figure 13:**
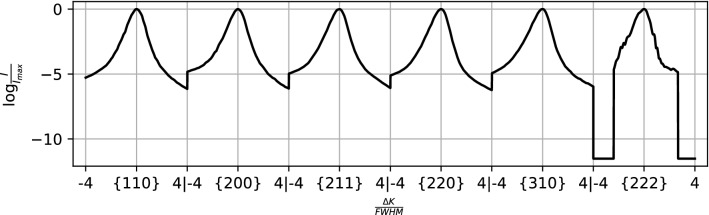
A processed profile taken from the set of experimentally obtained neutron diffraction profiles, here shown on a log scale. The peaks shown were processed to remove background intensity, then normalized with respect to maximum intensity and *FWHM*.

Each peak to be used in the surrogate model was extracted from the remaining noisy profile. A combination of an analytical function and the experiment data was used, i.e.,

24 $$I_{comb} = a I_{ex} + \left( {1 - a} \right) I_{fit}$$

25 $$a\left( {{\Delta }K} \right) = \min \left( {1,\frac{{10 I_{ex} \left( {{\Delta }K} \right)}}{{I_{ex,max} }}} \right)$$

26 $$I_{fit} \left( {{\Delta }K} \right) = D\left( {\frac{DIFC}{{K_{hkl} + {\Delta }K}} + \frac{DIFA}{{\left( {K_{hkl} + {\Delta }K} \right)^{2} }} + ZERO - T_{ph} \left( {d_{hkl} } \right)} \right) = D\left( {{\Delta }t} \right)$$

where $I_{ex}$ is the background subtracted peak from the experimental data, $I_{fit}$ is an analytical function fit to the experimental peak, and $I_{comb}$ is a combination of the two that is taken to be used with the surrogate model. The fitted function is defined using $D\left( {{\Delta }t} \right)$: a convolution of a gaussian, a split exponential, and a Lorentzian function, i.e.,

27 $$D\left( {{\Delta }t} \right) = L\left( {{\Delta t}} \right)*H\left( {{\Delta }t} \right)$$

28 $$L\left( {{\Delta }t} \right) = \frac{{l^{2} }}{{l^{2} + {\Delta }t^{2} }}$$

where $H$ is defined in Eq. (11) and $l$ is a fitting parameter. The function was fit to each peak using a least squares minimization. In effect, the tails were approximated using an analytical fitting function, while the experimental peak shape was preserved above a noise floor. An example processed experimental profile is shown in Fig. 13.

### eCMWP broadening model

In eCMWP, a diffraction peak is decomposed into Fourier coefficients to describe broadening in terms of multiple contributing factors: strain broadening, size broadening, and instrument broadening,

29 $$A_{L} \left( {\mathbf{K}} \right) = A_{L}^{D} \left( {\mathbf{K}} \right)A_{L}^{S} \left( {\mathbf{K}} \right)A_{L}^{I} \left( {\mathbf{K}} \right)$$

where $A_{L}^{D}$ are the strain broadening Fourier coefficients, $A_{L}^{S}$ denote size broadening, and $A_{L}^{I}$ denote the instrument broadening, all in terms of the Fourier length $L$. The definition of the instrument broadening can vary with experiment, and the form used in this work is described in “Virtual diffraction” section. Size broadening has a negligible influence on this dataset. The strain broadening is chiefly due to dislocations. These effects are calculated as,

30 $$A_{L}^{D} \left( {\varvec{K}} \right) = \exp \left( { - 2\pi^{2} L^{2} K^{2} \left[ {\left( {\frac{b}{2\pi }} \right)^{2} \pi \rho Cf\left( {\frac{L}{{R_{e}^{*} }}} \right)} \right]} \right),$$

with Burgers vector $b$, dislocation density $\rho$, contrast factor $C$, and Wilkens function $f$^31^. $R_{e}^{*}$ is a correlation length parameter, which describes the arrangement of dislocations. The strain field of a dislocation is anisotropic, and the strain that is apparent along each diffraction vector ${\mathbf{K}}$ will vary. The contrast factor $C_{hkl}$ quantifies the apparent strain of each dislocation type at each diffraction vector. Within eCMWP for this work, dislocations are assumed to be equally distributed among slip systems. An average contrast factor is calculated for each $hkl$^38,47,73^. For the cubic material, the average contrast factor $\overline{C}_{hkl}$ is calculated for each peak as,

31 $$\overline{C}_{hkl} = C_{h00} \left( {1 - q_{av} H^{2} } \right)$$

32 $$H^{2} = \frac{{h^{2} k^{2} + h^{2} l^{2} + k^{2} l^{2} }}{{\left( {h^{2} + k^{2} + l^{2} } \right)^{2} }}$$

where $q_{av}$ is the dislocation character parameter (determined by the proportion of screw and edge dislocations), and $C_{h00}$ is determined from dislocation crystallography and the elastic properties of the material.

## Data Availability

The data that support the findings of this study are available from the corresponding author upon reasonable request.
